# 4-Phenylbutyric Acid Induces Protection against Pulmonary Arterial Hypertension in Rats

**DOI:** 10.1371/journal.pone.0157538

**Published:** 2016-06-15

**Authors:** Yun Wu, Dilare Adi, Mei Long, Jie Wang, Fen Liu, Min-Tao Gai, Alidan Aierken, Ming-Yuan Li, Qian Li, Lei-Qi Wu, Yi-Tong Ma, Minawaer Hujiaaihemaiti

**Affiliations:** 1 Department of General Practice, First Affiliated Hospital of Xinjiang Medical University, Urumqi, 830011 P.R., China; 2 Xinjiang Key Laboratory of Cardiovascular Disease Research, First Affiliated Hospital of Xinjiang Medical University, Urumqi, 830011 P.R., China; 3 Department of Cardiology, First Affiliated Hospital of Xinjiang Medical University, Urumqi, 830011 P.R., China; 4 Department of Mechanism and Function, Xinjiang Medical University, Urumqi, 830011 P.R., China; 5 Department of Pharmacy, First Affiliated Hospital of Xinjiang Medical University, Urumqi, 830011 P.R., China; Vanderbilt University Medical Center, UNITED STATES

## Abstract

**Background:**

Endoplasmic reticulum (ER) stress has been implicated in the pathophysiology of various pulmonary diseases via the activation of the unfolded protein response. However, the role of ER stress in pulmonary arterial hypertension (PAH) remains unclear. The well-known chemical chaperone 4-phenylbutyric acid (4-PBA) inhibits ER stress signaling. We hypothesized that known chemical chaperones, including 4-PBA, would inhibit the activation of ER stress and prevent and/or reverse PAH.

**Methods and Results:**

Male Wistar rats were randomly divided into four groups: a normal control group (NORMAL group), a PAH group, and two PAH model plus 4-PBA treatment groups. The latter two groups included rats receiving 4-PBA by gavage each day as a preventive measure (the PRE group, with PBA starting on the day of PAH induction and continuing for 4 weeks) or as a reversal measure (the REV group, with PBA starting on the third week of PAH induction and continuing for 2 weeks). The PAH model was induced by intraperitoneally administering monocrotaline. The mean pulmonary artery pressure and mean right ventricular pressure were lower in the REV and PRE groups than in the NORMAL group. Furthermore, 4-PBA improved pulmonary arterial remodeling and suppressed the expression of ER stress indicators.

**Conclusion:**

Our findings indicate that PAH induces ER stress and provokes pulmonary arterial and right ventricular remodeling. Additionally, we show that attenuation of ER stress has the potential to be an effective therapeutic strategy for protecting pulmonary arteries.

## Introduction

Pulmonary arterial hypertension (PAH) is a refractory syndrome that causes restricted flow through the pulmonary arterial circulation. PAH can be associated with many conditions, including BMPR2 mutation, connective tissue diseases, portal hypertension, congenital heart disease and schistosomiasis. Because of the insidious onset of PAH, the median survival of untreated patients is limited to three years post diagnosis [1]. PAH is characterized by pulmonary vascular remodeling, including pulmonary artery smooth muscle cell (PASMC) proliferation, micro-thrombosis, and sustained pulmonary vasoconstriction. These conditions lead to increased pulmonary arterial pressure, right ventricular failure, and ultimately death [2]. Multiple pathogenic pathways have been implicated in the development of PAH, but their mechanisms remain unclear [3]. Maintaining a balance between vasoconstriction and vasodilation via administration of prostanoids, inhibition of PDE-5, and antagonism of endothelin receptors serves as a major therapeutic strategy [3]. However, there are no effective targeted therapies to restrain and reverse pulmonary arterial remodeling. Therefore, the prognosis for PAH remains poor, with a mortality rate of approximately 15% within one year of initiating treatment [4].

In recent studies, endoplasmic reticulum (ER) stress has been shown to play an important role in the development of PAH; ER stress results from unfolded and/or misfolded proteins being packaged in the ER [5, 6]. In the case of mild ER stress, the unfolded protein response (UPR) attempts to reduce the amount of misfolded proteins by increasing the production of ER chaperones such as GRP78 and GRP94, which optimize protein folding and maintain appropriate levels of Ca^2+^ and ATP [7]. However, persistent activation of the UPR leads to cellular dysfunction and apoptosis, which promote pulmonary arterial remodeling [8]. UPR results in cellular apoptosis by three known signaling pathways, which are triggered by activating transcription factor-6 (ATF6), inositol requiring enzyme-1 (IRE1), or PKR-like ER kinase (PERK) in the ER membrane [9]. A study from the Michelakis group introduced a new idea for the treatment of PAH. They observed that activation of ATF6 could lead to pulmonary vascular smooth cell proliferation; furthermore, PAH symptoms could be relieved by inhibiting ATF6 with chemical chaperones [8, 10]. However, whether the ATF6 branches is the only branch that is active, and what downstream molecular changes in the ATF6 branch are inhibited by chemical chaperones in ER stress, are unknown. It is also not well understood whether the PERK and IRE-1 branches of ER stress may be similarly affected by chemical chaperones during the development of PAH.

Sodium 4-phenylbutyric acid (4-PBA), a low-molecular-weight fatty acid, is an ammonia scavenger that is approved for clinical use in the treatment of pathological urea cycle disorders [11]. In addition, 4-PBA is a low-toxicity compound that protects against various noxious stimuli, and it can be orally administered. Therefore, it has been proposed as a treatment for sickle cell disease, cystic fibrosis, and cancer [12]. Importantly, 4-PBA can inhibit ER stress as a chemical chaperone by stabilizing peptide structures, improving the luminal folding capacity, and attenuating cell damage [8, 13, 14]. In this study, we aimed to investigate whether the attenuation of ER stress is able to protect against pulmonary arterial remodeling using a monocrotaline (MCT)-induced PAH rat model.

## Materials and Methods

### Ethics statement

All experiments were performed with the approval of the animal ethics committee of the First Affiliated Hospital of Xinjiang Medical University in accordance with the Care and Use of Laboratory Animals guidelines of the National Institutes of Health (permit number: IACUC-20140313005).

### Animals and experimental protocol

Male Wistar rats, weighing 250-280g, were randomly divided into four groups, as follows: a control group (NORMAL), a PAH group (PAH), a prevention group (PRE), and a reversal group (REV). PAH was induced by intraperitoneally administering MCT (Sigma-Aldrich; 60 mg/kg) once, on the first day of the experiment, as previously described [15, 16, 17]. Rats in the NORMAL group received the same volume of saline. The PAH rats were further randomly divided into three subgroups: (1) in the PAH group, rats were only observed; (2) in the REV group, PAH rats received 4-PBA (Sigma-Aldrich) starting on the third week of PAH induction and continuing for two weeks; and (3) in the PRE group, PAH rats received 4-PBA starting on the day of PAH induction and continuing for four weeks. The 4-PBA was administered daily by gavage at a dose of 500 mg/kg body weight in the REV and PRE groups. After four weeks, all animals were sacrificed using carbon dioxide asphyxiation followed by cervical dislocation. Lung and heart samples were removed and rapidly washed in cold 0.9% saline. The lung tissues were flash frozen in liquid nitrogen and stored at -80°C until they were used for real-time PCR and western blot analysis. Transection tissues drawn from the right hilum were stored in 10% formalin for hematoxylin-eosin staining.

### Hemodynamic parameter testing

After four weeks, rats were anesthetized by intraperitoneal injection of 1% sodium pentobarbital (45 mg/kg). Next, right ventricular pressure and pulmonary arterial pressure were measured using the BL-420F biological and functional information collection system (TME, Chengdu, China).

### Assessment of pulmonary arterial and right ventricular remodeling

After the hemodynamic parameters were tested and the animals were sacrificed, the excised lung and heart were divided. Next, the right ventricular free wall (RV) and the left ventricular plus septal (LV + S) wall were separated, washed, dried and weighed. The right ventricular hypertrophy index (RVHI) was calculated as [RV / (LV + S)], yielding a right ventricular remodeling parameter in PAH.

After being embedded in paraffin, samples were sliced and stained with hematoxylin-eosin. For each lung sample, the pathological morphology and the pulmonary arteriole of each group were randomly selected and observed under a light microscope. Image J software (National Institutes of Health, USA) was used to measure medial wall thickness (thickness between internal and external elastic plates, MWT), external diameter (the diameter of the external elastic plates, ED), vascular lumen area (VA), vascular wall area (WA), and total vascular area (TA) of the pulmonary arteriole membrane (diameters between 50–100 μm). The percentage of MWT (MWT% = 2 × MWT / ED), the ratio of WA/TA (WA%), and the ratio of VA/TA (VA%) were calculated to evaluate pulmonary arterial remodeling.

### Quantitative real-time PCR analysis

Total RNA was extracted using Trizol reagent according to the product protocol (TIANGEN, Beijing, China). For reverse transcription, 1 μg of total RNA was converted to first-strand cDNA in a 10-μl reaction mixture using the PrimeScript™ RT reagent Kit with gDNA Eraser (Takara Bio, Dalian, China). Quantitative real-time PCR analysis was performed using an ABI 7500 fluorescence quantitative PCR instrument (Applied Biosystems) using the 2-ΔΔCT method. The thermal cycling program began with 30 s at 95°C for enzyme activation and then included 45 cycles of denaturation for 5 s at 95°C and annealing and extension for 40 s at 60°C. Primer sequences are listed in Table 1 (Invitrogen, Beijing, China).

**Table 1:** Primers used in quantitative real-time PCR assays.

Genes	Forward primers (5’->3’)	Reverse primers (5’->3’)	Expected size (bp)
GRP94	GAGGCTGAATCTTCTCCGTTTG	CTGTTGCTTCCCGACTTTCTTTAC	193
GRP78	GAATCCCTCCTGCTCCCCGT	TTGGTCATTGGTGATGGTGATTTTG	134
ATF6	TGCAGGTGTATTACGCTTCG	CTTCGGTCTTGTGGTCTTGTTA	146
IRE	CATCCGAATGTGATCCGCTAC	CCTTCTGCTCCACATACTCCTG	106
PERK	TTCCGAAGCCACCTTGTCTACC	ACTCTGTGCTCCCTGTCCTCCAT	195
CHOP	CTTCACTACTCTTGACCCTG	TGAGCCATAGAACTCTGACTGGAATC	120
Bcl-2	GGGCTACGAGTGGGATACTGGAG	CGGGCGTTCGGTTGCTCT	101
GAPDH	TGGAGTCTACTGGCGTCTT	TGTCATATTTCTCGTGGTTCA	138

### Western blot analysis

To assess protein levels, total protein was extracted from stored lung tissues. Next, protein extracts were separated by 10% SDS-PAGE and transferred to polyvinylidene difluoride (PVDF) membranes (pore diameter 0.22 μm) (Millipore, USA). The membranes were blocked at room temperature for 1 h with a blocking solution (3% BSA) in TBST buffer (10 mM Tris, 100 mM NaCl, 0.1% Tween 20, pH 7.4). The blots were incubated overnight at 4°C with primary antibodies, including anti-ATF6 (abcam^®^, cat. no. ab37149, Cambridge, MA, USA), anti-GRP78 (ab188878), anti-GRP94 (ab3674), anti-PERK (ab115617), anti-BCL-2 (ab18210) and anti-CHOP (cat. no. 15204-1-AP, ProteinTech Group, Inc, Chicago, IL, USA). The membranes were then washed in TBST buffer 3 times for 10 minutes each. Bound primary antibodies were incubated with horseradish peroxidase-labeled secondary antibodies for 1 h at room temperature, washed, and detected using ECL. Densitometry was conducted with a UVP gel imaging system (USA). Blots were stripped and re-probed with a β-actin antibody to confirm equal loading.

### Statistical analysis

All data are expressed as mean ± SEM or as a percentage. Differences between groups were assessed using one-way ANOVA followed by LSD post hoc analysis (least significant difference, LSD) for multiple comparisons. P<0.05 was considered to be statistically significant.

## Results

### 4-PBA decreases pulmonary arterial and right ventricular pressure

**Fig 1:**
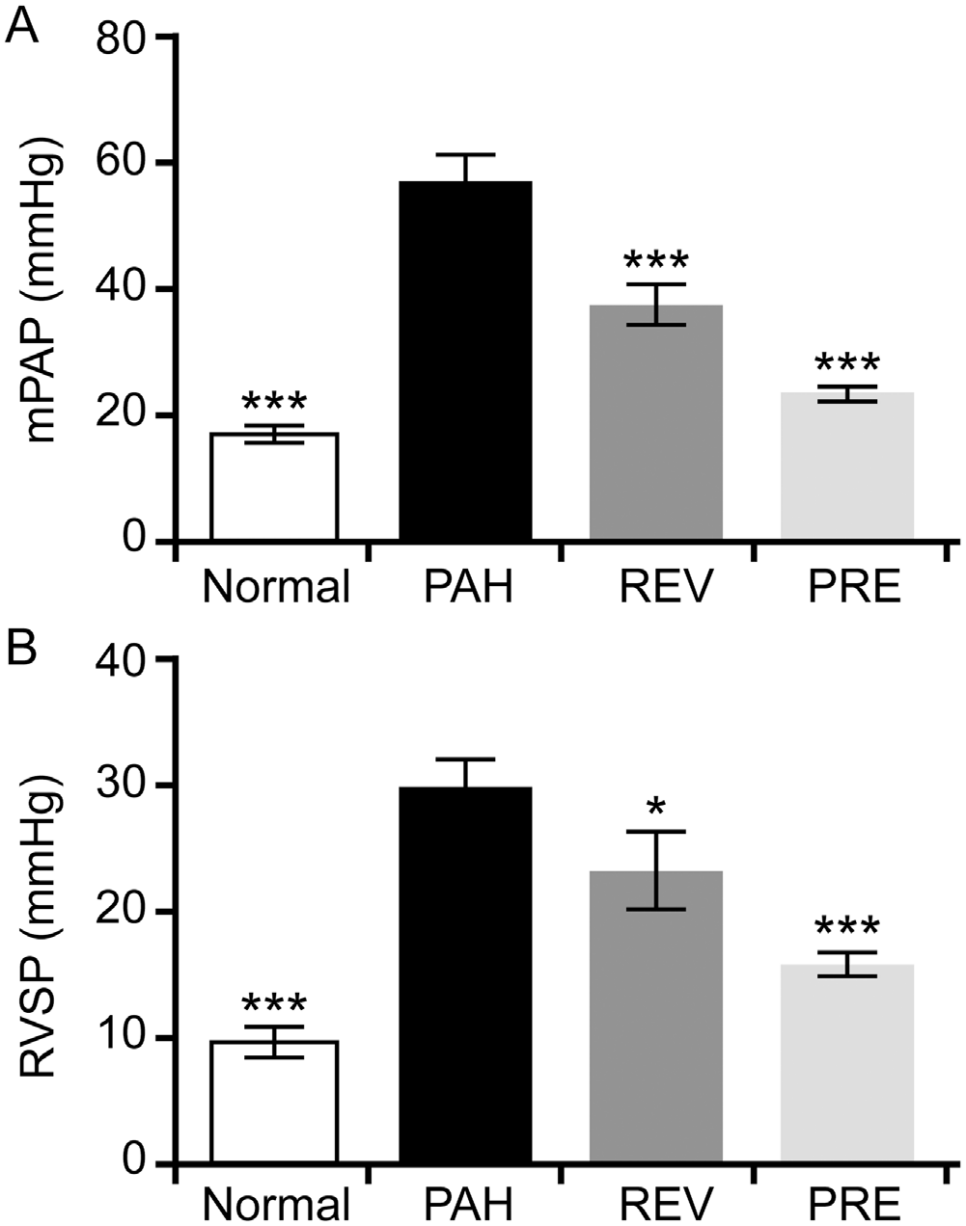
Effects of 4-PBA on mean pulmonary arterial pressure (mPAP) and right ventricular pressure (RVSP) in a rat model of MCT-induced pulmonary arterial hypertension. (A) mPAP (mmHg) observed in rats in the NORMAL (n = 12), PAH (n = 6), REV (n = 8) and PRE (n = 8) groups. (B) RVSP (mmHg) observed in rats in the NORMAL (n = 12), PAH (n = 6), REV (n = 9) and PRE (n = 8) groups. Compared to the MCT-induced PAH model group, significant changes in mPAP and RVSP were observed for all other groups. *p < 0.05; **p < 0.01; ***p < 0.001.

To determine if 4-PBA can protect against PAH, we measured hemodynamic parameters, including mean pulmonary arterial pressure (mPAP) and right ventricular pressure (RVSP) in rats. The changes in hemodynamic parameters from survival at day 28 are shown in Fig 1. Rats in both 4-PBA treatment groups had lower mPAP values than rats in the PAH group (37.28 ± 3.96 mmHg in the REV group and 23.18 ± 1.34 mmHg in the PRE group versus 56.68 ± 4.47 mmHg in the PAH group). Furthermore, mPAP in the REV group (23.18 ± 1.34 mmHg) showed values similar to rats in the NORMAL group (17.07 ± 1.43 mmHg) (Fig 1A). Additionally, rats treated with 4-PBA in either group had lower RVSP values than rats in the PAH group (22.76 ± 3.47 mmHg and 15.38 ± 1.04 mmHg for the REV and PRE groups, respectively, versus 29.51 ± 2.52 mmHg; Fig 1B).

### 4-PBA attenuates pulmonary arterial and right ventricular remodeling

**Fig 2:**
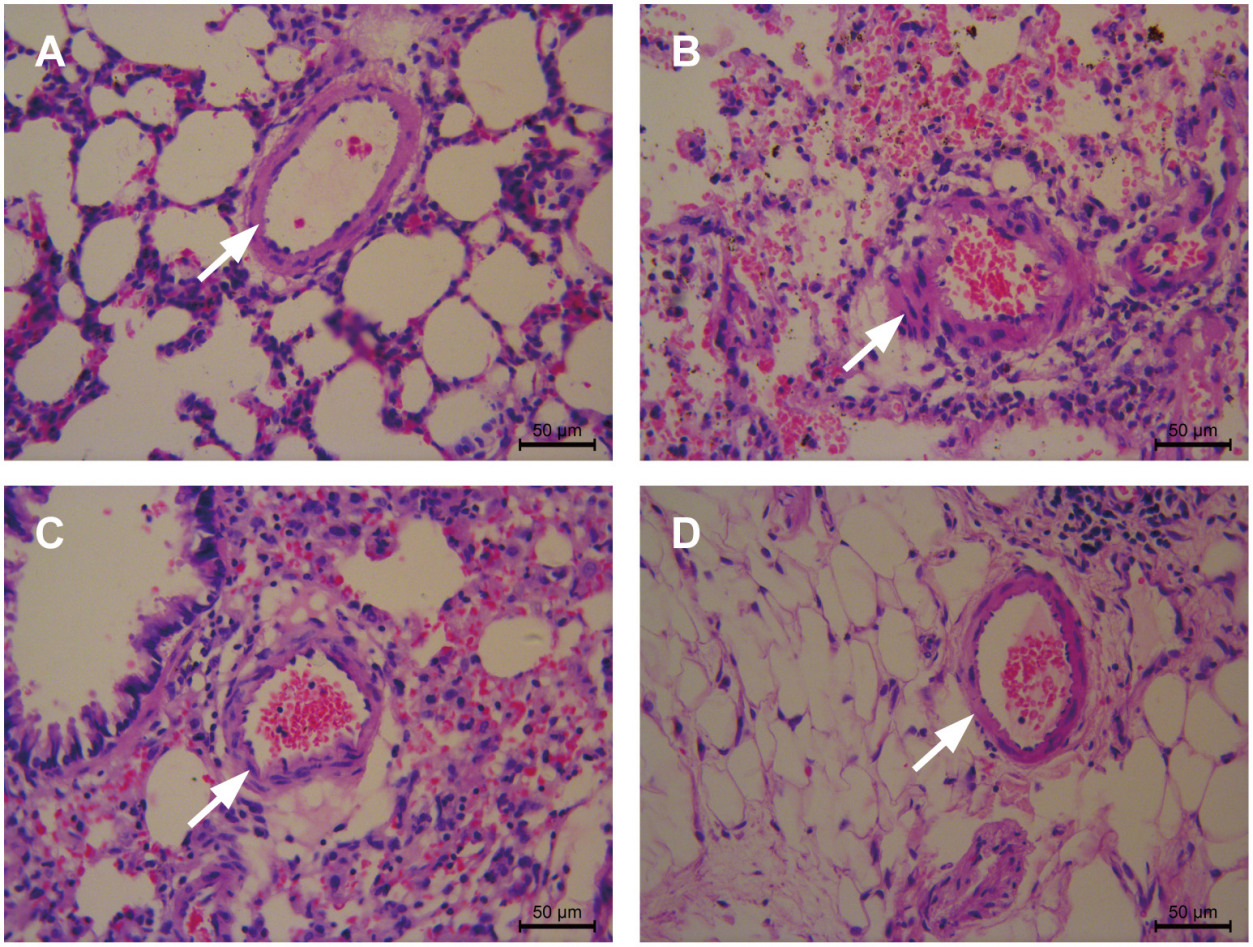
4-PBA attenuates pulmonary arterial remodeling. Lung histology examples from the NORMAL group (A), PAH group (B), REV group (C) and PRE group (D) are shown. In the NORMAL group, the thickness of the pulmonary arterioles was normal and the distribution of smooth muscle cells was even. There was no congestion in the lumen of the pulmonary artery. In the PAH group, significantly increased pulmonary arterial wall thickness, luminal congestion, interstitial lung hemorrhage and increased inflammatory cell infiltration were observed, (marked by arrows). In the REV group, the MCT-induced pulmonary arterial wall thickening and inflammatory cell infiltration were significantly attenuated by 4-PBA. In the PRE group, compared with the PAH group, significantly decreased pulmonary arterial wall thickness, no luminal congestion, and less inflammatory cell infiltration were observed. Scale bars = 50 μm.

**Fig 3:**
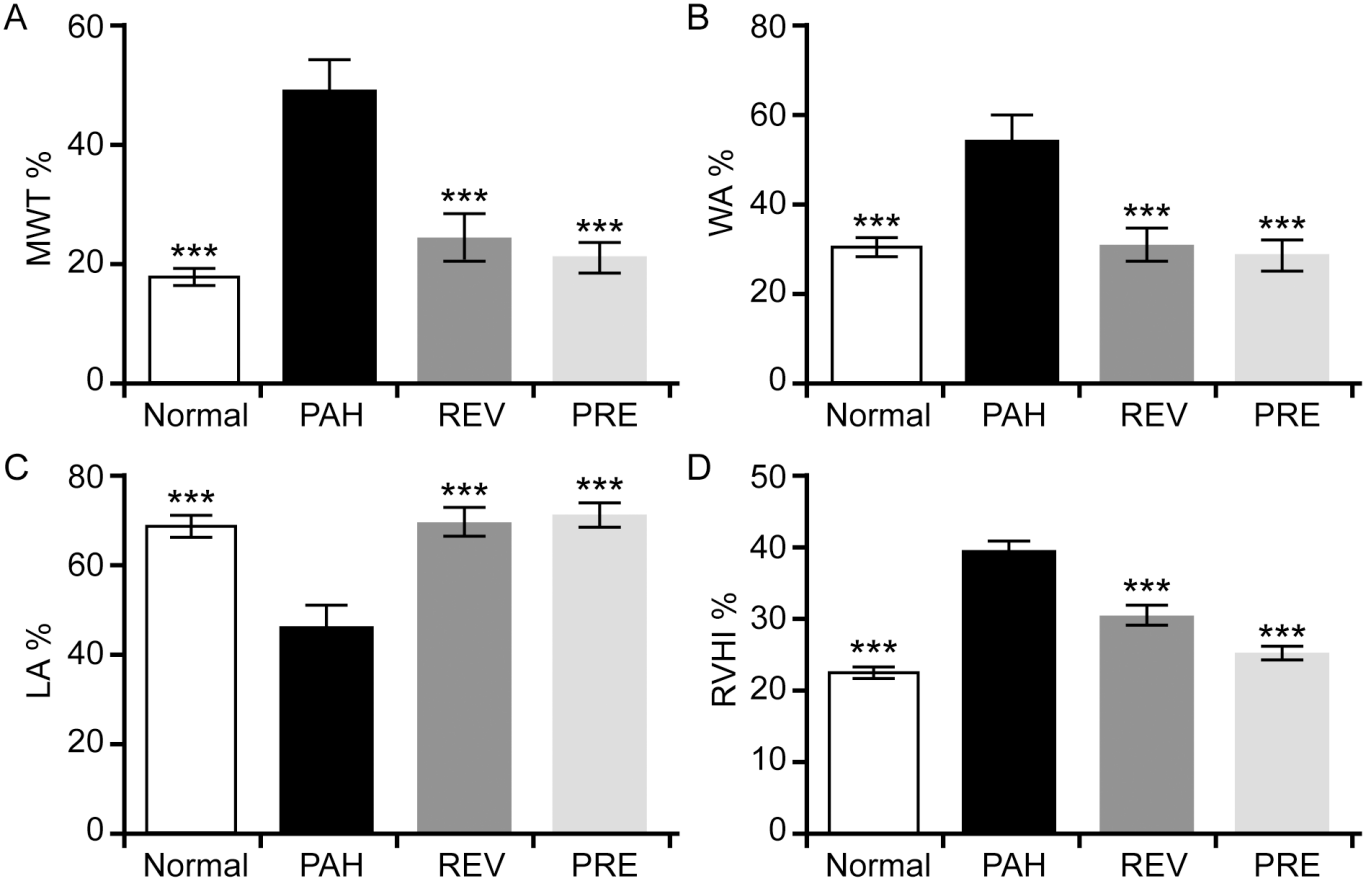
4-PBA reverses and prevents pulmonary arterial and right ventricular remodeling in a rat model of MCT-induced PAH. The percent medial wall thickness (MWT%, A), the ratio of vascular wall area (WA) / total vascular area (WA%, B), the ratio of vascular lumenal area (VA) / total vascular area (luminal erea, LA%, C) and the right ventricular hypertrophy index (RVHI%, D) were examined in the NORMAL, PAH, REV and PRE groups (n = 10 for each group). Compared to the PAH group, significantly lower MWT%, WA% and RVHI% values were observed in the other groups (A, B and D), while significantly higher LA% values were observed (C). ***p < 0.001.

Lung histology in the PAH group showed smooth muscle cell proliferation, uneven cell distribution, increases in pulmonary arterial wall thickness, increased luminal stenosis, and a glut of inflammatory cells when compared with that of the NORMAL group. Consistent with the changes in hemodynamic parameters, the changes in pulmonary arterial remodeling were clearly attenuated with 4-PBA treatment (Fig 2). The MWT% and WA% in the treated groups were significantly lower than in the PAH group, and the VA% in the treated group was higher than in the PAH group (Fig 3A, 3B and 3C). A clear decrease in the RVHI implied that right ventricular hypertrophy was prevented by the addition of 4-PBA (Fig 3D). There were no statistically significant differences in the histology parameters among the NORMAL, REV and PRE groups.

### 4-PBA inhibits ER stress markers in PAH

**Fig 4:**
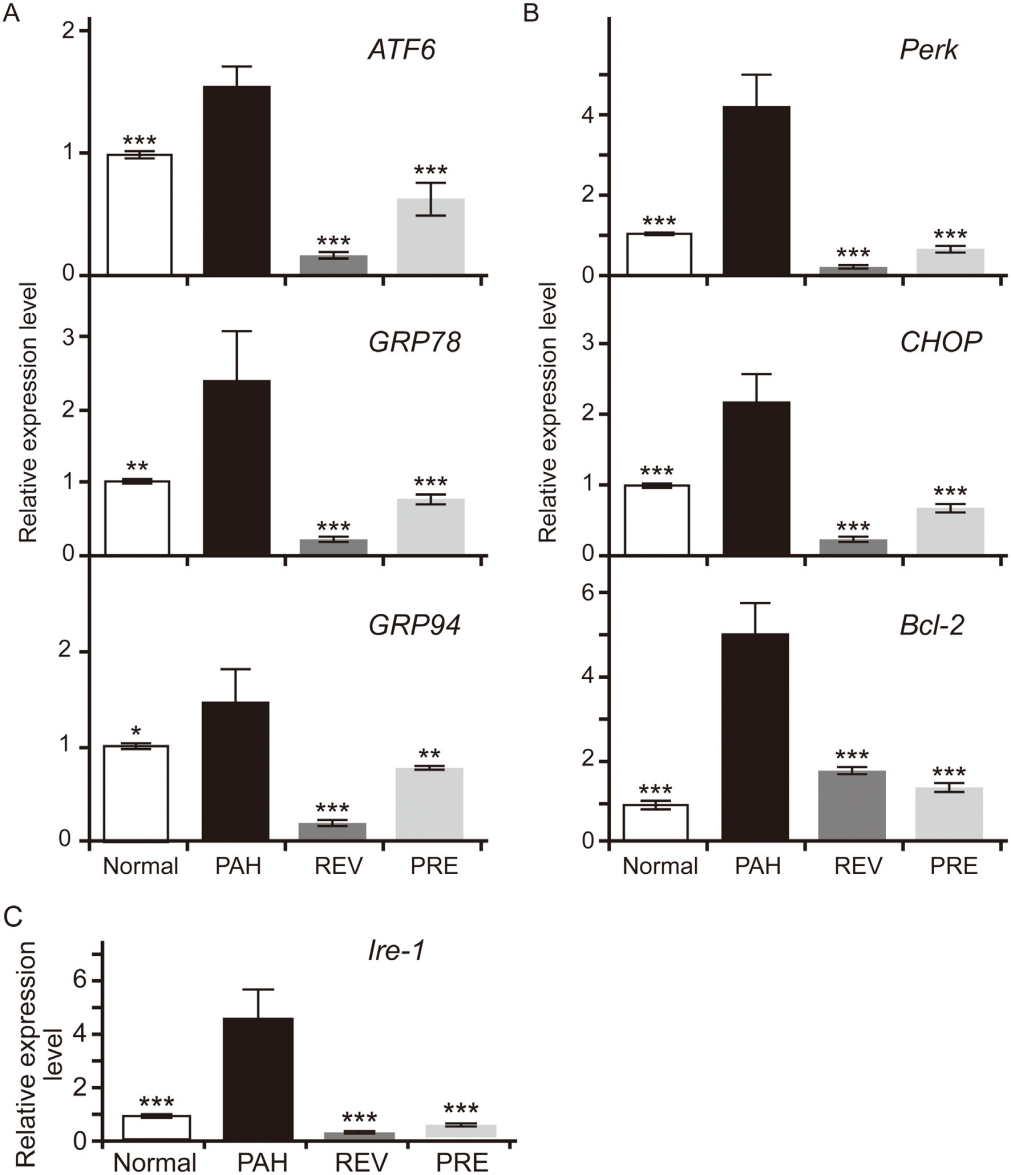
Effects of 4-PBA on gene expression levels in the main branches of the ER stress signaling pathway in MCT-induced PAH lungs. (A) Relative expression levels of three representative genes in the ATF6 branch of ER stress. *ATF6*, activating transcription factor-6; *GRP78*, 78-kDa glucose-regulated protein; *GRP94*, 94-kDa glucose-regulated protein. (B) Relative expression levels of three representative genes in the PERK branch of ER stress. *PERK*, PKR-like ER kinase; *CHOP*, transcription factor C/EBP homologous protein; *BCL-2*, apoptosis regulator Bcl-2. C. Relative expression levels of three representative genes of the IRE-1 branch of ER stress. *IRE-1*, inositol-requiring enzyme-1. In each group, n = 10. For each gene, the expression level of the NORMAL group was set to 1. Compared to the PAH group, significant changes in expression levels were found. *p < 0.05; **p < 0.05; ***p < 0.001.

In this study, we examined whether the ATF6 branch is a positive regulator of ER stress in PAH rats. In addition to ATF6 being an ER stress sensor, GRP78 and GRP94 are upstream targets of the ATF6 branch (Fig 4A). CHOP and BCL-2, which are downstream targets of ER stress, were used to assess the activation of ATF6 in the ER stress signaling pathway. The expression of several genes, including GRP78, GRP94, ATF6, IRE-1, PERK, CHOP, and BCL-2, were upregulated in the PAH group but were inhibited in the two treatment groups (Fig 4B and 4C). The mRNA levels of GRP78, IRE-1, Perk and BCL-2 were not significantly different among the NORMAL, REV, and PRE groups, while the GRP94 and CHOP mRNA levels were not significantly different between the PRE and NORMAL groups.

**Fig 5:**
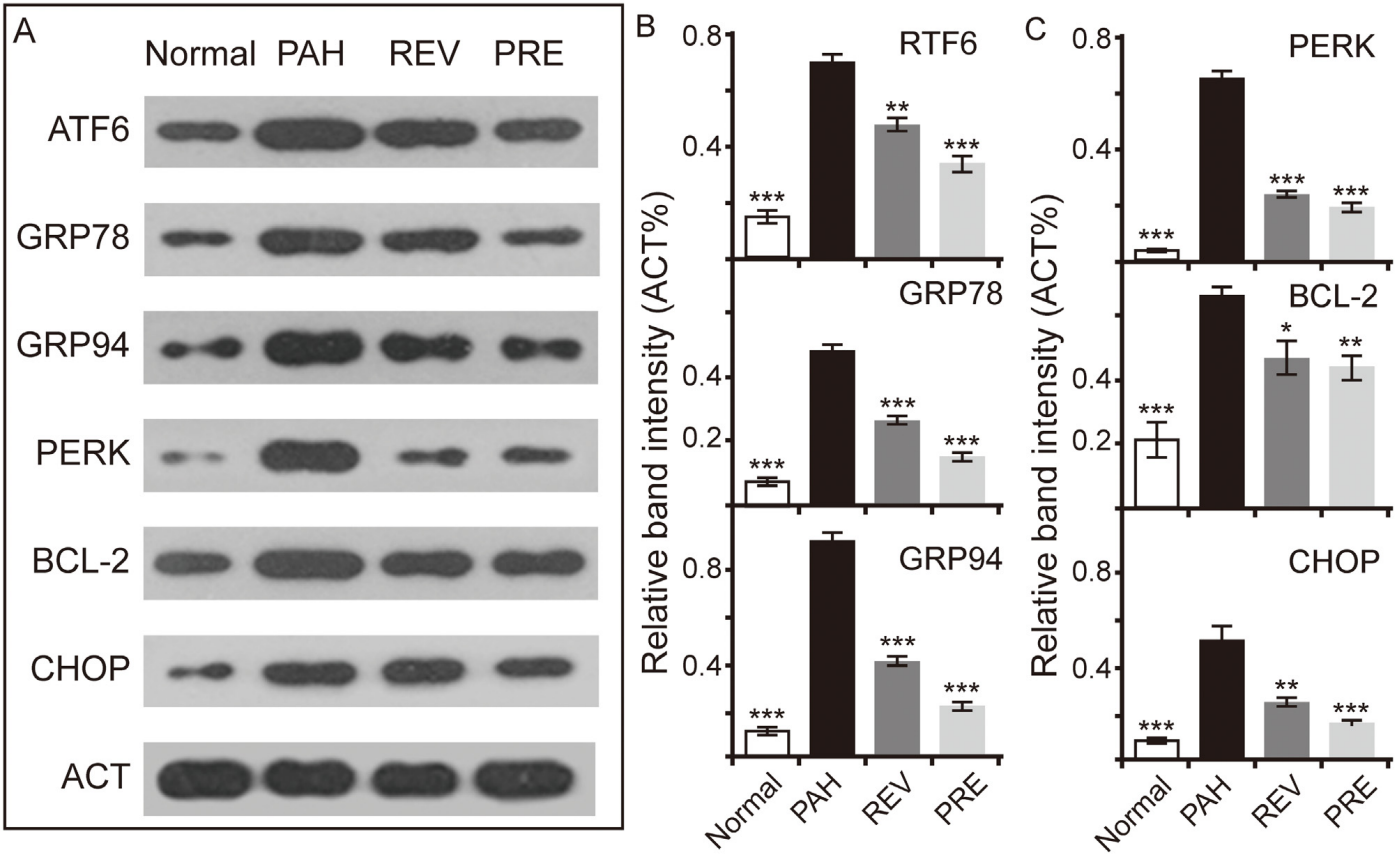
Effects of 4-PBA on protein levels in the ATF6 and PERK branches of ER stress signaling in MCT-induced PAH lungs. (**A)** Western blots of proteins in the ATF6 and PERK branches of ER stress. β-actin (ACT) was used as a housekeeping protein. (B) Relative band intensities of three proteins in the ATF6 branch of ER stress (compared to ACT). (C) Relative band intensities of three proteins in the PERK branch of ER stress (compared to ACT; n = 3 for each group). Statistical analysis was performed exactly as in Fig 4.

Consistent with the mRNA data, increased protein levels of GRP78, GRP94, ATF6 (the 50-kDa fragment was detected), PERK, CHOP, and BCL-2 were observed in the PAH group (Fig 5A). Evaluation of the protein band intensities revealed that the gray value in the PAH group was significantly higher than that in the NORMAL group (P < 0.001). However, protein expression decreased in the treatment groups in comparison with the PAH group (Fig 5B). In addition, the protein levels of PERK, CHOP and BCL-2 were not significantly different between the REV and PRE groups (Fig 5C). As downstream targets of ATF6 branch, the changes in CHOP and BCL-2 levels may imply that 4-PBA treatment for PAH in the REV group can achieve the same effects as observed for the PRE group. Additionally, the non-significant changes observed in the protein levels of PERK, the key molecule of the PERK branch, in the REV and PRE groups may also imply that the ATF6 branch is not the only pathway that is affected by 4-PBA.

## Discussion

In the present study, we hypothesized that inhibition of ER stress by 4-PBA would alleviate pulmonary arterial remodeling in rats with PAH. The data from our study indicate that mPAP and RVSP decrease under 4-PBA treatment and that the primary molecules of ER stress are suppressed by 4-PBA. These observations are consistent with previous studies [8, 10]. In a study by the Michelakis group, it was reported that 4-PBA inhibited the ATF6 signaling pathway by suppressing Nogo, a member of the reticulum family of proteins, in hypoxia-induced and MCT-induced PAH. In the present study, we observed that 4-PBA inhibited not only Nogo but also other key molecules in the ATF6, IRE-1 and PERK branches of ER stress in MCT-induced PAH.

The ER is the largest intracellular membrane network structure. The ER has two primary functions, namely the synthesis and processing of proteins and cellular signal processing [18–21]. Often, nascent polypeptides are folded and assembled to form active proteins and specific spatial structures in the ER. Multiple molecular chaperones, including calnexin, the heavy chain binding protein BiP, GRP78, GRP94, and others are involved in this process [22–25]. There are also three ER membrane sensors: ATF-6, IRE-1, and PERK [8, 10, 26, 27]. When the body is in a state of stress, GRP78 combines these sensors on the cytoplasmic side of the ER, dissociates from the ER, and further activates the UPR [28]. ER stress is characterized by changes in specific protein expression, including a lower translation rate, ER chaperone protein upregulation, and misfolded protein degradation. These changes can successfully restore ER homeostasis. ER stress is an important cellular defense and is evolutionarily conserved. However, persistent or strong ER stress can also trigger apoptosis. Myriad factors, including ischemia-reperfusion injury, oxidative stress, homocysteine, excessive intracellular protein synthesis capacity, and other synthetic barriers or physical, chemical, and genetic factors can induce ER stress, ultimately leading to apoptosis [29–32]. CHOP (transcription factor C/EBP homologous protein) a transcription factor that controls genes encoding components involved in apoptosis, is specifically expressed under conditions of ER dysfunction [23]. Our results showed that CHOP was expressed at low levels in the NORMAL group but was upregulated at the mRNA and protein levels in rats in the PAH group, suggesting that ER stress occurs during development of PAH and may further induce apoptosis. This result was in accord with those of Koyama M [13], but it differed from those of the Michelakis group [8], which showed that CHOP expression decreased in chronic hypoxia-induced PAH in mice. This discrepancy may relate to oxygen concentration and experimental duration. In the MCT-induced PAH model, MCT activates ER stress mainly by stimulating inflammation. In other drug-induced inflammatory animal models, CHOP was expressed at low levels in the control group and was upregulated by inflammation [33, 34]. However, these studies showed that CHOP is not an independent and necessary factor for inducing apoptosis during ER stress.

Interestingly, we found that the level of BCL-2 increased in MCT-induced PAH, in contrast to certain previous results [23, 35]. However, other studies observed the upregulation of BCL-2 in pulmonary hypertension [36, 37]. Endothelial cell apoptosis plays an important role at the early stages of PAH. At later stages of PAH, excessive endothelial cell proliferation, vasoconstriction, micro-thrombosis, and vascular remodeling are important pathological changes that have been verified by period studies [38]. At least 20 BCL-2 family members have been identified in mammalian cells, and several others have been identified in viruses. These members are divided into two categories, including anti-apoptotic and pro-apoptotic subfamilies. BCL-2 is a member of the anti-apoptotic subfamily [39]. At the early stages of PAH, BCL-2 levels decrease and endothelial cell apoptosis increases, so that the level of BCL-2 is lower than normal. However, at later stages of PAH, a higher level of BCL-2 is observed in PAH patients and animal models, which is associated with excessive endothelial cell proliferation [36, 37, 40].

During ER stress, free IRE-1 and PERK are activated by dimerization and phosphorylation, respectively, within their cytoplasmic domains. In addition, ATF6 is hydrolyzed by a protease in the Golgi apparatus to become an active transcription factor. Next, the transcription of CHOP is induced; CHOP accumulates in the nucleus and promotes apoptosis. During ER stress, PERK is considered to be activated first and IRE1 last [26, 27, 41–43]. In our study, we observed that all of the three main branches of ER stress signaling could be activated in our MCT-induced PAH animal model and that all of the branches could be inhibited by 4-PBA. This finding may indicate that ER stress is a ubiquitous protection mechanism, and it also implies that the selective targets of 4-PBA have been uncertain.

The compound 4-PBA is an FDA-approved drug that is primarily used for the treatment of urea cycle disorders. Studies have shown that 4-PBA acts as a chemical chaperone, as an ammonia scavenger and a weak histone deacetylase (HDAC) inhibitor. Several studies have suggested that 4-PBA acts as a molecular chaperone to prevent protein aggregation and aids in protein folding in the ER [11–14]. In the present study, we observed that 4-PBA can significantly reduce effector molecule expression in the ATF6 branch of ER stress signaling. GRP78 is an initial factor in ER stress, and it is a valid indicator for the activation of the ER stress signaling pathway. When the expression of GRP78 is suppressed, ER stress is blocked accordingly. Besides ATF6 suppression, the expression of CHOP, a command executor in the ER stress pathway, also was reduced in PBA-treated groups. Unlike previously reported results [8], the function of 4-PBA was non-specific, affecting the ATF6, IRE-1, and PERK branches of ER stress in our experiments.

## Study limitations

This study evaluated the effect of a chemical chaperone on PAH. We observed that 4-PBA downregulated several key proteins in ER stress. However, ER stress is a ubiquitous protection mechanism. How may a specific target be selected? How are pro-survival and pro-apoptotic pathways in ER stress defined? What is the best clinical application of 4-PBA in PAH patients? There are several issues yet to be resolved, and we need to investigate the key targets in ER stress.

## Conclusion

Although ER stress has not been considered to be a key factor in the development of PAH, our study implies that attenuation of ER stress could be an effective therapeutic strategy for the protection of pulmonary arteries. The chemical chaperone 4-PBA can significantly decrease mPAP and improve pulmonary arterial and right ventricular remodeling.
